# The beta cell glucocorticoid receptor protects against hyperglycaemia by modulating insulin secretion during glucocorticoid rhythm disruption in mice

**DOI:** 10.64898/2026.06.03.730005

**Published:** 2026-06-08

**Authors:** Jijo Wilson, Anna Arzeno, Sanjeev Sharma, Agnes Agas, Jack Lungstrum, Mary N. Teruel

## Abstract

**Aims/hypothesis:**

Disruption of the circadian glucocorticoid rhythm occurs in human settings including chronic stress, sleep restriction, circadian misalignment, ageing and autonomous cortisol secretion; in mild autonomous cortisol secretion (MACS) and Cushing’s syndrome, loss of the normal cortisol trough is clinically informative, and flatter diurnal cortisol profiles are associated with cardiometabolic disease. We previously showed that flattening of glucocorticoid rhythms in mice induces rapid and sustained hyperinsulinemia without hyper or hypo-glycaemia, implying that glucocorticoid rhythms may directly regulate the relationship between circulating glucose and systemic insulin output. Here we tested the hypothesis that beta cell glucocorticoid receptor (GR) signalling is required for the compensatory hyperinsulinaemia that maintains glucose homeostasis during glucocorticoid rhythm flattening, and that this reflects glucocorticoid-dependent reprogramming of beta cell stimulus-secretion coupling.

**Methods:**

Glucocorticoid rhythms were flattened in male C57BL/6J mice by subcutaneous corticosterone pellet implantation, which elevates trough levels and reduces peak amplitude while preserving the daily mean hormone concentration. Fasting plasma insulin and blood glucose were measured longitudinally and compared with placebo-implanted controls and high-fat diet–fed mice. Beta cell secretory function was assessed by static and dynamic glucose-stimulated insulin secretion in isolated islets, and beta cell excitability by GCaMP6f Ca²⁺ imaging in islets from Ins1-Cre;GCaMP6f mice. To test the requirement for beta cell GR in mature beta cells while avoiding developmental effects of constitutive GR deletion, we generated adult-inducible beta cell–specific GR knockout mice (MIP-CreERT;Nr3c1fl/fl; βGRKO). Combined beta cell and hepatic GR knockout mice (double-GRKO) were used to examine an additional extra-pancreatic contribution to systemic insulin availability. Glucose tolerance and insulin sensitivity were assessed by intraperitoneal glucose and insulin tolerance tests. As a secondary question, a possible contribution of altered insulin clearance was examined from plasma C-peptide:insulin ratios and hepatic insulin-degrading enzyme (IDE) abundance.

**Results:**

Glucocorticoid rhythm flattening produced sustained hyperinsulinaemia with maintained euglycaemia, distinct from the delayed hyperinsulinaemia and hyperglycaemia observed in high-fat diet–fed mice. Islets from glucocorticoid-flattened mice exhibited increased insulin secretion at subthreshold (3 mmol/l) glucose, enhanced secretory responses to stimulatory glucose and increased Ca²⁺ responses, indicating a lowered glucose threshold for beta cell activation that persisted ex vivo. Beta cell–specific deletion of GR markedly attenuated the hyperinsulinaemic response to glucocorticoid flattening (insulin AUC reduced ∼40% vs controls; p < 0.001) and produced progressive hyperglycaemia and impaired glucose tolerance, despite unchanged or improved insulin sensitivity. A reduced plasma C-peptide:insulin molar ratio (p = 0.007) and decreased hepatic IDE abundance (p = 0.032) indicated that reduced insulin clearance contributes additionally to the elevated circulating insulin, and combined beta cell and hepatic GR deletion lowered circulating insulin further than beta cell GR deletion alone. The absence of hypoglycaemia despite persistent hyperinsulinaemia is consistent with concurrent insulin resistance.

**Conclusions/interpretation:**

Beta cell GR signalling is required for the compensatory hyperinsulinaemia that maintains glucose homeostasis when glucocorticoid rhythmicity is disrupted, acting through glucocorticoid-dependent lowering of the glucose threshold for insulin secretion; reduced insulin clearance contributes additionally to the rise in circulating insulin. These findings identify beta cell GR signalling as a key determinant of glucose homeostasis during disrupted glucocorticoid rhythmicity. Clinically, the work is most relevant not simply to nonspecific chronic stress, but to human states in which the cortisol rhythm is measurably flattened or the nocturnal trough is lost, including MACS, Cushing’s syndrome, sleep restriction, shift work/circadian misalignment and ageing.

**RESEARCH IN CONTEXT:**

**What is already known about this subject?**

- Flattened or disrupted glucocorticoid rhythmicity in humans is observed most directly in MACS and Cushing’s syndrome, where loss of the late-night cortisol nadir is clinically informative, and more broadly as flatter salivary cortisol slopes or elevated evening cortisol in ageing, sleep restriction and circadian misalignment; these patterns are associated with type 2 diabetes, cardiovascular disease and mortality.
- Flattening of glucocorticoid rhythms in mice induces rapid and sustained hyperinsulinaemia without hypoglycaemia, indicating that circulating insulin can be elevated independently of glucose.
- Hepatic insulin clearance, mediated in part by insulin-degrading enzyme and CEACAM1, is a major determinant of circulating insulin levels.

**What is the key question?**

- How does disruption of glucocorticoid rhythmicity increase circulating insulin while maintaining glycaemic control, and is beta cell glucocorticoid receptor signalling required for this adaptive response?

**What are the new findings?**

- Glucocorticoid rhythm flattening lowers the glucose threshold for beta cell activation through enhanced Ca²⁺ excitability, an effect that persists in isolated islets and indicates *in vivo* reprogramming of beta cell function.
- Beta cell–specific deletion of the glucocorticoid receptor blunts the hyperinsulinaemic response to glucocorticoid flattening and produces hyperglycaemia and impaired glucose tolerance despite unchanged or improved insulin sensitivity.
- Reduced insulin clearance, associated with decreased hepatic insulin-degrading enzyme abundance, contributes additionally to the elevated circulating insulin, but is not required for maintenance of glucose homeostasis.

**How might this impact on clinical practice in the foreseeable future?**

- Identifying beta cell glucocorticoid receptor signalling as a requirement for glucose homeostasis during disrupted glucocorticoid rhythmicity may inform strategies for understanding hyperinsulinaemia and steroid-associated metabolic dysfunction in human conditions marked by loss of the cortisol trough or flatter diurnal cortisol profiles, particularly MACS, Cushing’s syndrome, shift work/circadian misalignment and ageing.

## Full Text Availability

The license terms selected by the author(s) for this preprint version do not permit archiving in PMC. The full text is available from the preprint server.

